# Late-life fasting imparts resiliency and protein persulfidation

**DOI:** 10.18632/aging.203758

**Published:** 2021-12-13

**Authors:** Jie Yang, Betemariam Sharew, Christopher Hine

**Affiliations:** 1 Department of Cardiovascular and Metabolic Sciences, Cleveland Clinic Lerner Research Institute, Cleveland, OH 44195, USA; 2 Cleveland Clinic Lerner College of Medicine, Cleveland, OH 44195, USA

**Keywords:** Every-other-Day (EOD) fasting, hydrogen sulfide (HS) _2_, cystathionine γ-lyase (CGL), persulfidation

Declines in physical, cognitive, and metabolic faculties are common during the process of aging. While there is no shame in these manifestations; as nature forever marches to the beat of its drum in all of us, it is encouraging to recognize scientific and medical innovations have uncovered means to battle the erosion of ourselves. While these interventions are not the miracle elixirs once touted by traveling medicine men and provide nothing resembling immortality, they do offer potential to add healthy years, delay the onset of frailty, and stave off aging-related diseases such as cancer, cardiovascular disease, inflammation, and metabolic syndrome.

These interventions in pre-clinical studies largely focus on pharmacological, genetic, regenerative, and dietary means to extend healthy lifespan. Regenerative approaches involve the removal of senescent cells combined with engineered replacement for aging reversal. The other three geroscience approaches largely slow inherent aging rates. Numerous drugs impacting nutrient sensing, metabolism, and antioxidant defense systems extend healthy lifespan in experimental organisms across evolutionary boundaries. Genetic models of mammalian longevity include disruption to growth hormone/IGF-1 or mTOR signaling. Dietary restriction, which includes caloric restriction, intermittent fasting, and methionine restriction, are the oldest and best studied interventions to extend longevity [1]. A variety of mechanisms are proposed that deliver the benefits of these geroscience approaches, with enhanced expression of hepatic cystathionine γ-lyase (CGL) a shared hallmark of all three [2]. CGL is involved in transsulfuration and produces the redox modifying gasotransmitter hydrogen sulfide (H_2_S) which functions through protein persulfidation and mitochondrial metabolic regulation [3].

A question in the field regarding effective clinical translation of these interventions is at what age are they to be applied? Starting prior to ones mid-20s could have detrimental impacts on development, growth, and reproductive capacity. As aging slowly begins in ones 30s and then rapidly accelerates in the 6^th^ and 7^th^ decades, it seems fitting to commence during this timeframe. However, is it ever too late to start? And if so, what is the point where these interventions no longer work, or worse, negatively impact quality of life? Likewise, do interventions started late in life still activate CGL and H_2_S production/signaling as possible mechanisms of action?

**Figure 1:**
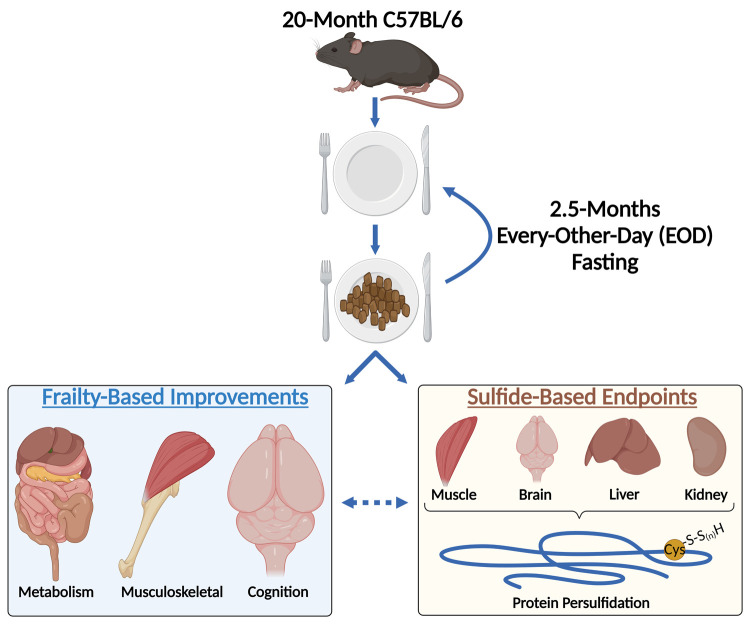
**Late-life intermittent fasting improves cognitive and physiological abilities concurrent with multi-tissue protein persulfidation in a sex-dependent manner.** 20-month old C57BL/6 mice were placed on 2.5-months of Every-Other-Day (EOD) fasting. During and at the end of this intermittent fasting period, performance in metabolic, musculoskeletal, and cognitive tests were measured. Male mice improved in these areas, while little to no improvement was observed in female mice. Mechanistic examination to changes in hydrogen sulfide-related biology triggered by the EOD fasting revealed augmented protein persulfidation in muscle, brain, liver, and kidney. The dotted line between the two outcomes suggests a connection between decreased frailty and augmented H_2_S production and signaling. Figure created with BioRender.com.

Human studies of scale needed to answer these questions are difficult to rigorously and reliably perform. Alternatively, laboratory models in rodents provide testable and mechanistic platforms to examine means of slowing the mammalian aging. In two companion studies published this year [4,5], our lab sought to determine if the dietary intervention of every-other-day (EOD) fasting applied at 20-months of age in C57BL/6 mice, which is approximately 60-65 years for humans, could improve physical, cognitive, and metabolic abilities. We discovered 2.5 months of EOD fasting reduced overall caloric intake, hypothalamic inflammatory gene expression, and frailty in male, but not female, mice. Likewise, musculoskeletal, hippocampal-dependent cognitive, and metabolic endpoints were improved in these aged male mice undergoing EOD fasting [4]. In examining H_2_S-related changes the aged mice after the 2.5 months of EOD fasting, we uncovered augmented renal H_2_S production capacity which positively correlated with improvements in the frailty tasks [4]. Evaluation of multi-organ protein persulfidation via a biotin-thiol assay coupled with mass spectrometry analysis revealed late-life EOD fasting increased protein persulfidation in liver, kidney, skeletal muscle, and brain, albeit not always to the same extent imparted by mid-life dietary interventions [5]. Interestingly and still not completely understood, we found late-life EOD fasting decreased protein persulfidation in the heart, which was also observed under mid-life dietary interventions [5].Thus, EOD fasting when started relatively late in life still improved overall health of male study subjects and trigged similar CGL/H_2_S responses as in younger cohorts (Figure 1).

What are the next scientific and clinical steps to be taken based on this work? Many questions remain regarding mechanism, timing, and translational efficacy. Investigation into why EOD fasting failed to improve aging female mice would be an interesting avenue to approach. This sex-dependent outcome could be due to multiple reasons, one being female mice overcompensated food intake on fed days, resulting in no net-loss caloric consumption [4]. Thus, integration of both EOD fasting combined with forced caloric restriction on the fed day could provide insight. Moreover, EOD fasting concomitantly decreases vitamin and mineral intake such as iron. Maintenance of iron and haem metabolism play causal roles in aging. Thus, sex-specific differences in iron metabolism, which could impact non-enzymatic H_2_S production [6], may explain sex differences in response to EOD fasting. Alternatively, the gut microbiome make-up, its related metabolites, and how they change during EOD fasting have not been extensively investigated as mechanisms for improved lifespan or shifts in host CGL activity and H_2_S signaling. Fasting may impact microbiome populations of sulfate reducing bacteria such as *Desulfovibrio piger,* which ultimately increase host H_2_S levels [7]. Likewise, it is unknown if other microbiome-related metabolites altered during fasting regulate host H_2_S production. Lastly, integration of late-life intermittent fasting into the clinic may not be ready for widespread adoption for lifespan extension. However, on an individual basis and in consultation with a healthcare provider, based on our preclinical results and the preliminary clinical studies of others [8], it may be a viable lifestyle change providing tangible benefits including reduced adiposity, managed blood pressure, and physical, metabolic, and cognitive improvements.
